# Controlled Coherent Coupling in a Quantum Dot Molecule Revealed by Ultrafast Four-Wave Mixing Spectroscopy

**DOI:** 10.1021/acsphotonics.3c00108

**Published:** 2023-05-08

**Authors:** Daniel Wigger, Johannes Schall, Marielle Deconinck, Nikolai Bart, Paweł Mrowiński, Mateusz Krzykowski, Krzysztof Gawarecki, Martin von Helversen, Ronny Schmidt, Lucas Bremer, Frederik Bopp, Dirk Reuter, Andreas D. Wieck, Sven Rodt, Julien Renard, Gilles Nogues, Arne Ludwig, Paweł Machnikowski, Jonathan J. Finley, Stephan Reitzenstein, Jacek Kasprzak

**Affiliations:** † Institute of Theoretical Physics, Wrocław University of Science and Technology, 50-370 Wrocław, Poland; ‡ School of Physics, Trinity College Dublin, Dublin 2, D02 PN40, Ireland; § Institute of Solid State Physics, Technische Universität Berlin, 10623 Berlin, Germany; ∥ Lehrstuhl für Angewandte Festkörperphysik Ruhr-Universität Bochum, 44780 Bochum, Germany; ⊥ Laboratory for Optical Spectroscopy of Nanostructures, Department of Experimental Physics, Wrocław University of Technology, 50-370 Wrocław, Poland; # Walter Schottky Institut and Physik Department, Technische Universität München, 85748 Garching, Germany; ○ Department Physik, Universität Paderborn, 33098 Paderborn, Germany; □ Université Grenoble Alpes, CNRS, Grenoble INP, Institut Néel, 38000 Grenoble, France

**Keywords:** quantum dot molecule, nonlinear spectroscopy, coherent control, quantum coherence, light−matter coupling, four-wave mixing

## Abstract

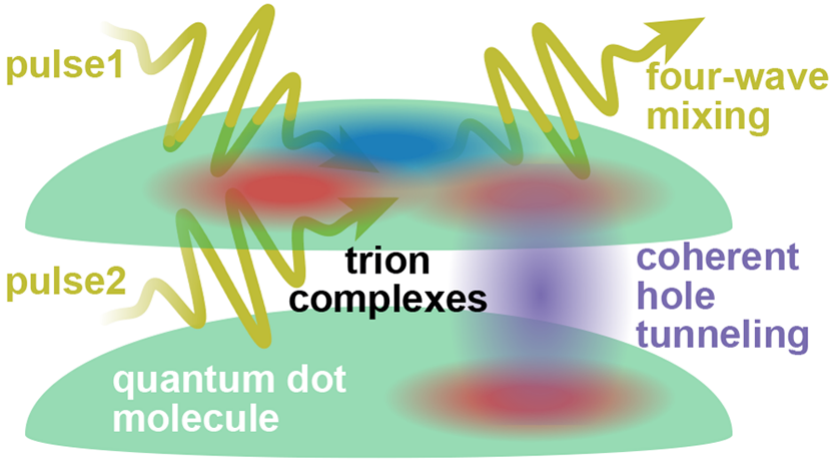

Semiconductor quantum dot molecules are considered promising candidates for quantum technological applications due to their wide tunability of optical properties and coverage of different energy scales associated with charge and spin physics. While previous works have studied the tunnel-coupling of the different excitonic charge complexes shared by the two quantum dots by conventional optical spectroscopy, we here report on the first demonstration of a coherently controlled interdot tunnel-coupling focusing on the quantum coherence of the optically active trion transitions. We employ ultrafast four-wave mixing spectroscopy to resonantly generate a quantum coherence in one trion complex, transfer it to and probe it in another trion configuration. With the help of theoretical modeling on different levels of complexity, we give an instructive explanation of the underlying coupling mechanism and dynamical processes.

## Introduction

Maintaining and controlling coherence of quantum states spanning multiple physically distinct systems is at the core of quantum technologies, like quantum information processing and communication^1^ or quantum metrology.^2^ In the competitive and multidisciplinary quest for systems that might allow one to efficiently induce, control, and detect quantum coherence, exciton complexes in semiconductor quantum dots (QDs) were already recognized very early as promising building blocks.^3,4^ QDs offer an efficient light–matter interface,^5^ which makes it possible to control their quantum states at subpicosecond time scales,^6^ beyond cryogenic temperatures up to ambient conditions.^7^

Closely stacked QDs forming quantum dot molecules (QDMs) offer a large flexibility to control their optical and spin properties by external electric or magnetic fields.^8−13^ Therefore, they are particularly attractive for quantum applications, such as quantum repeaters, requiring efficient spin–photon interfaces. While spectral characteristics of the coupling have been revealed in QDM systems by linear spectroscopy methods,^8−14^ these experiments are only sensitive to the structure of eigenstates and their occupations, thus yielding a typical spectral picture that essentially characterizes two coupled modes of any kind, be it classical or quantum. In contrast, tracing coherences, which are fingerprints of quantum superpositions, requires the application of a coherent nonlinear spectroscopy tool, like four-wave mixing (FWM). This poses a major challenge, in particular in the case of single quantum systems, where the optical signal is very weak. For this reason, detecting coherent characteristics of the coupling between different excitonic complexes in self-assembled QDs is still in its infancy.

Fortunately, the epitaxial growth and nanoprocessing of QD-based quantum devices has been improved close to perfection, and individual nanostructures can nowadays be embedded deterministically into advanced nanophotonic devices with high photon extraction efficiency to improve the application potential and to enable experiments relying on high single-photon flux.^15,16^ On the other hand, the development of heterodyning and interferometric techniques in FWM spectroscopy made it possible to apply this method to single quantum emitters and to detect and characterize the coupling between individual transitions in various systems.^17−23^

Here, we go significantly beyond experiments on basic single structures by using QDMs with a much richer excitonic level scheme that can be controlled by an external bias. This allows us to demonstrate that the hole-tunnelling in the QDM can be used to transfer resonantly created quantum coherence between different trion transitions. Similar experiments were previously performed on quantum wells.^24−27^ In contrast to those, a single QDM is an atomic-like system, which offers broader perspectives for quantum technologies, e.g., interfacing spins and photons, at the same time making the experiment much more challenging. This way we demonstrate the coherent control of coherences in a coupled QD system, as required for quantum applications.^6^ We further show how this coherent coupling can be controlled by applying an electric field. In quantum optical terms, this amounts to parametrically switching between a V-system and a Λ-system in a single physical structure, which allows us to address different pairs of coherences.

Through this first demonstration, we make a crucial step toward the implementation of the ultrafast FWM methodology in photonic quantum technologies. In this way our work paves the way toward the realization of controlled long-range coherent coupling between distant solid state qubits.

## Device and Experimental Method

**Figure 1:**
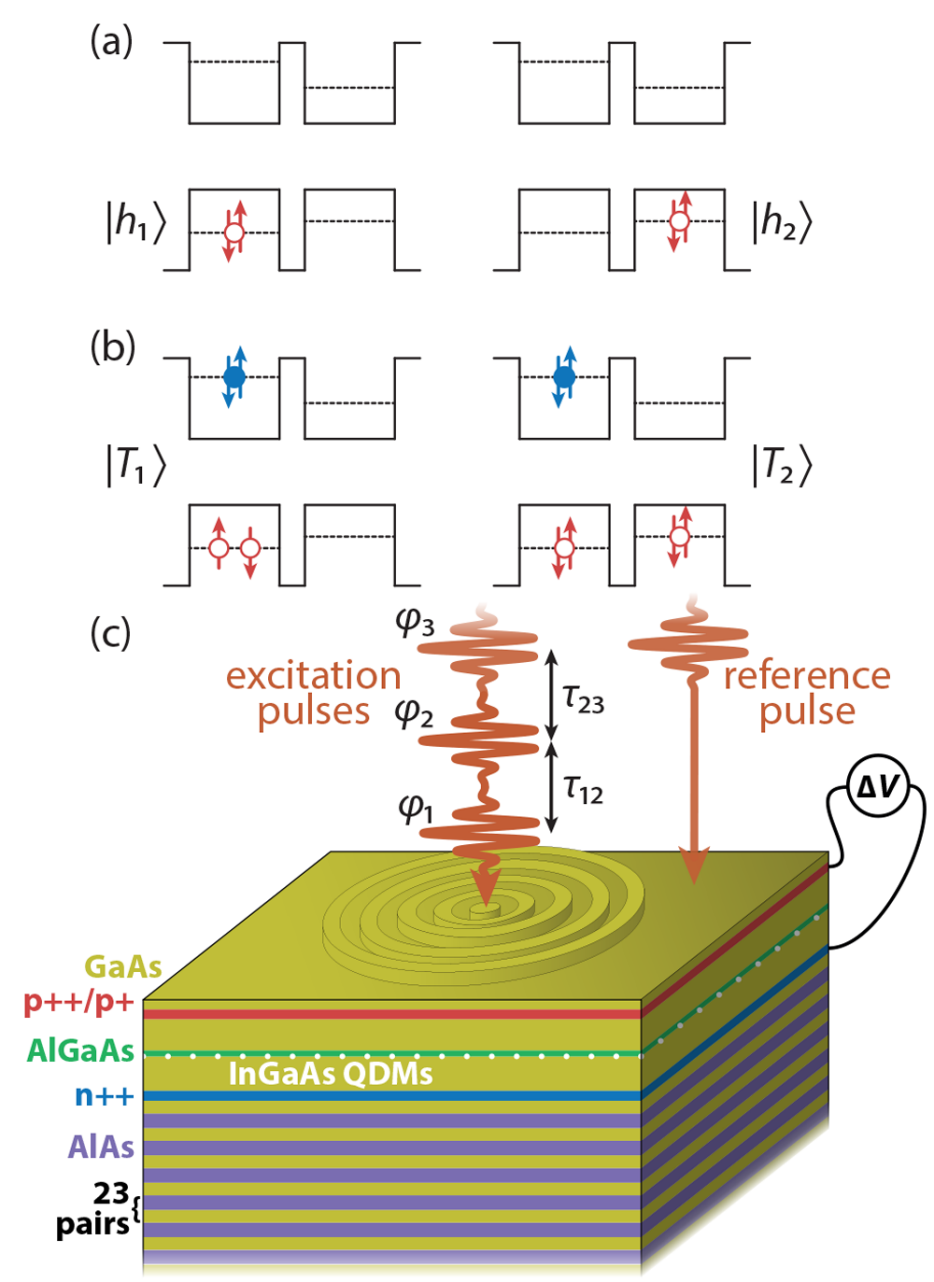
Involved QDM states and experimental setup. (a) Ground states of the involved trions possessing one hole in either of the quantum dots. (b) Relevant excited states formed by an additional exciton in the left quantum dot. (c) Schematic picture of the sample structure. Above a GaAs/AlAs distributed Bragg reflector the InGaAs quantum dot molecule (QDM) layer is sandwiched between charge-doped layers, which are connected to a bias source. The optical in- and out-coupling is improved by a circular Bragg grating, also called a bulls-eye photonic structure, on the top surface. The laser pulses used for the optical excitation in the four-wave mixing experiment are focused on the center of the grating, while the reference beam hits the free surface in the vicinity.

We here use the aforementioned advantages of semiconductor quantum photonic technology to demonstrate controlled coherent coupling between excitonic transitions hosted by a pair of InAs QDs separated by ≈8 nm, forming a QDM.^8,9,14,16^ The system is doped so that, apart from spin degeneracy, the ground state manifold of the QDM consists of two states, with a hole in one or the other QD, as schematically depicted in Figure 1a. As seen in the sketch in Figure 1b, from the two coupled charged three-particle complexes (trions) that can be excited optically, one is entirely located in one of the QDs, while the other spans both dots. We prove the coupling between the optically induced coherences between these hole and trion states in the coupled QDM system by performing two-dimensional four-wave mixing (2D FWM) spectroscopy (see below), in which coherent coupling between different optically active transitions is revealed as off-diagonal peaks in the 2D spectra.^23^ Similar results can be expected for exciton transitions in the other QD, which are typically separated by several meV and are therefore not accessible in this study due to the limited spectral width of the excitation.^13^

In our sample, the QDMs are placed in the structure schematically shown in Figure 1c, the layer structure of this QDM quantum device supports storage of holes by an AlGaAs tunneling barrier between the QDMs and the p-doped region of the diode. To achieve suitably high optical in- and out-coupling efficiency for the FWM signal,^21^ a circular Bragg grating is deterministically positioned around the QDM in the center of the quantum device. For details on the sample design and processing we refer to ref (16). The charge-doped layers surrounding the QDM sheet provide a p-i-n diode structure allowing voltage control. While scanning the external bias voltage, we monitor the formation of off-diagonal peaks in 2D FWM spectra of the molecule.

## Results

### Photoluminescence Spectra

**Figure 2:**
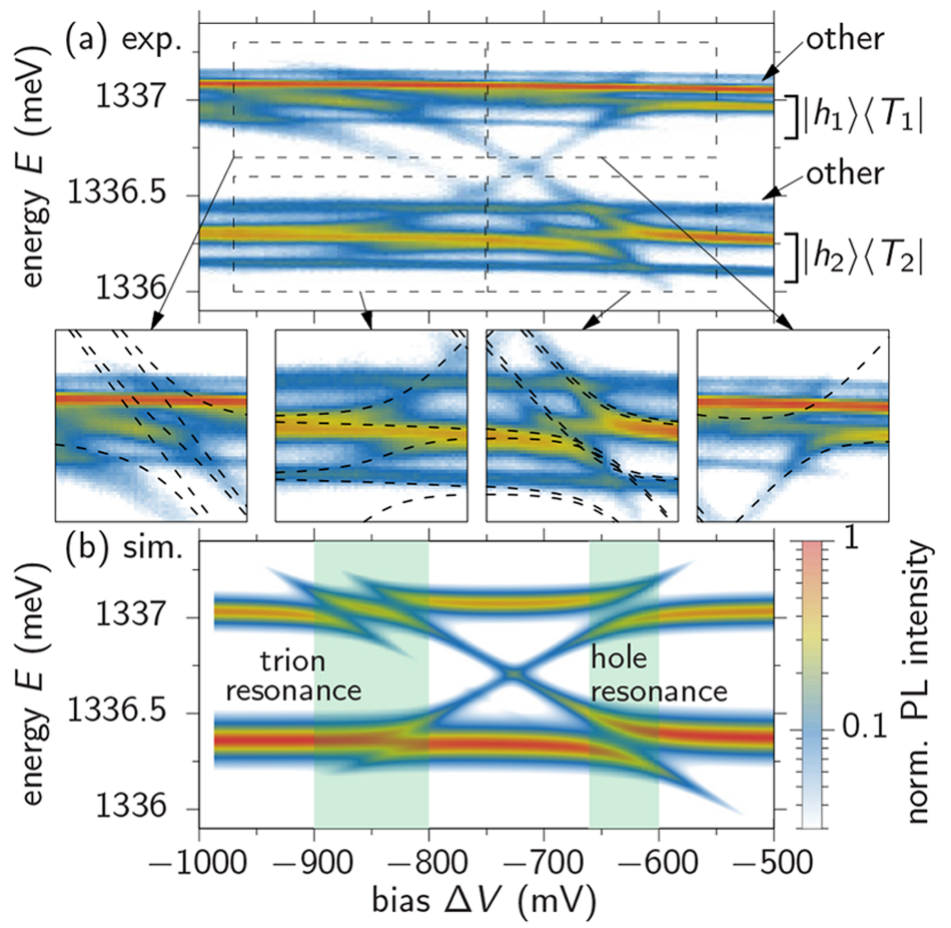
Bias scan of the PL spectra of the QDM-device. (a) Measured PL spectra as a function of the applied bias. The labels on the right mark the respective trions that correspond to Figure 5a and transitions from other exciton complexes, likely neutral excitons. The zoom-ins show the calculated optical transitions as dashed lines. (b) Semiempirical simulation of the PL spectrum. The avoided crossings marked by the green shaded areas on the left stem from trion (excited state) resonances and those on the right from hole (ground state) resonances.

For characterizing the QDM’s optical properties we measure photoluminescence (PL) spectra for varying applied electric fields, which leads to an energy shift of the involved electronic states with respect to each other. Consequently, the hole and trion states in the two dots are brought into resonance for specific electric fields and the tunnel-coupling leads to characteristic avoided crossings in the optical spectra. The results of this precharacterization are depicted in Figure 2a and are in excellent agreement with the findings in ref (16). To further check the integrity of the emitter structure we calculate the transition energies for a heuristic, semiempirical model of the QDM as shown by the dashed lines (details are given in the Supporting Information (SI)). We use the same fitting parameters as in ref (16) and achieve the same high level of agreement with the measured spectral lines. Note that not all possible transitions from the rich spin configurations including singlet and triplet states predicted by the theory (dashed lines) appear as bright spectral lines in the measurement because their dipole matrix elements are small and the optical signal is not visible at the given signal-to-noise ratio. While the entire structure of the PL spectra is quite complex with a total of ten visible lines, we are particularly interested in the four pronounced avoided crossings, which indicate state hybridizations and coherent tunnel-couplings. Two avoided crossings appear around an applied bias of Δ*V* ≈ −650 mV and are attributed to the resonance between the two hole states (ground states). The other two at Δ*V* ≈ −850 mV stem from the resonance of the trion states (excited states). We also find other bright spectral lines that are unaffected by the applied electric field and do not participate in the avoided crossings (marked by “other”). These transitions likely belong to neutral excitons in the QDM (SI).

In Figure 2b we show simulated PL spectra derived from a semiempirical model^28,29^ (SI) leading to the dashed lines in the zoom-ins in (a). Due to the optical selection rules, we find that not all transitions lead to bright emission lines in the spectrum (SI). The brightest lines, which form avoided crossings, are well reproduced by the simulation. In order to relate the spectra to the morphological characteristics of the QDM and to confirm the form of the spectrum with a model based on realistic wave functions and an extended number of hole and trion states, we have performed ***k***·***p*** calculations^30−41^ of the trion PL spectra. In spite of the multiple transitions present in the ***k***·***p*** model, the low-excitation PL spectrum is very similar to that obtained in the semiempirical model (SI).

Although the line splittings in the PL spectra certainly stem from the coupling between the two different exciton complexes, the appearance of an avoided crossing can be described already by a classical coupled oscillator model. Therefore, our goal is to directly address quantum coherence properties of the coupling mechanism via FWM microspectroscopy. In this experiment the QDM is resonantly excited with three laser pulses  (see Figure 1c and SI). Each of the pulses is phase-labeled by , which can experimentally be achieved by different propagation directions ***k***_1,2,3_(42) or, as in our case, by radio frequency-shifts (Ω_1_, Ω_2_, Ω_3_) = (80, 79, 79.77) MHz.^17^ The considered FWM signal is then carried by the phase combination φ_3_ + φ_2_ – φ_1_,^43,44^ which means that, at small pulse areas, we are detecting the third-order nonlinearity  (χ^(3)^ regime). This FWM signal from the sample is retrieved via a heterodyne detection with a reference pulse that is focused next to the grating structure on the sample surface (Figure 1c). As explained in more detail below, the first pulse resonantly creates trion quantum coherences, which are transferred to the other trion states by tunnel-coupling and the second and third pulse. The FWM signal is then emitted with respectively different energies. By varying the delay between the first and second pulse τ_12_ and keeping τ_23_ = 0 fixed, we are able to probe the coherence dynamics in the system (SI). Note that this method directly probes the quantum coherence between the involved optically active states and therefore immediately demonstrates the quantum nature of the observed avoided crossing. Besides the coherent coupling, this spectroscopy method goes well beyond conventional (μPL) spetroscopy and can give valuable insight in the homogeneous broadening,^45,46^ phonon-induced dephasing processes,^47^ or Rabi oscillations.^43^ Moreover, by changing the pulse powers, we can characterize the light–matter coupling strength^48^ (SI).

### Four-Wave Mixing Spectra

**Figure 3:**
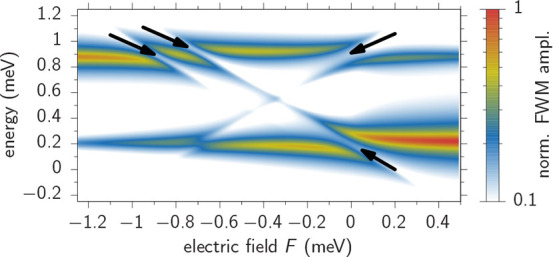
Simulated bias scan of the FWM spectrum of the QDM corresponding to the dashed lines in Figure 2a. The arrows mark the clear avoided crossings.

To get a first expectation for the avoided crossing behavior in FWM, we apply the model used to describe the PL spectra in Figure 2 and calculate the FWM spectra for ultrafast laser pulses in the δ-pulse limit depicted in Figure 3, where we consider vanishing pulse delays τ_12_ = τ_23_ = 0 and a dephasing rate of γ = 0.05 meV. Note that the electric field is here given as an energy shift between the lowest hole states in the two dots. We also calculated corresponding spectra by solving the Lindblad equation considering a nonvanishing pulse duration of Δ*t* = 0.2 ps and achieved a very similar result (SI). Here, we find the same structure of avoided crossings as marked by the arrows. The lower energy one at *F* ≈ – 0.75 meV is bridged by a spectral line, such that one avoided crossing remains at *F* = 0 meV. In the higher energetic transitions we find three clear gaps in the spectrum (arrows), two around *F* ≈ – 0.75 meV and the other at *F* = 0 meV. In addition, the intensity distribution of the different lines has to be addressed. Due to the thermal occupation (*T* = 7 K, as in the experiment) of the two ground (hole) states before any optical excitation, the resulting intensities depend on which of the QDs has the lowest hole state energy for a given applied electric field. This finally results in brighter FWM lines at lower energies for larger bias values and in higher intensities at larger transition energies for smaller bias values. The visibility of the crossing lines around *F* = −0.4 meV (Δ*V* ≈ – 730 mV in Figure 2b) is significantly reduced in FWM compared to PL (Figure 2b). This happens because of the nonlinear character of the signal in the χ^(3)^ regime, which suppresses transitions with weaker dipole matrix elements.

**Figure 4:**
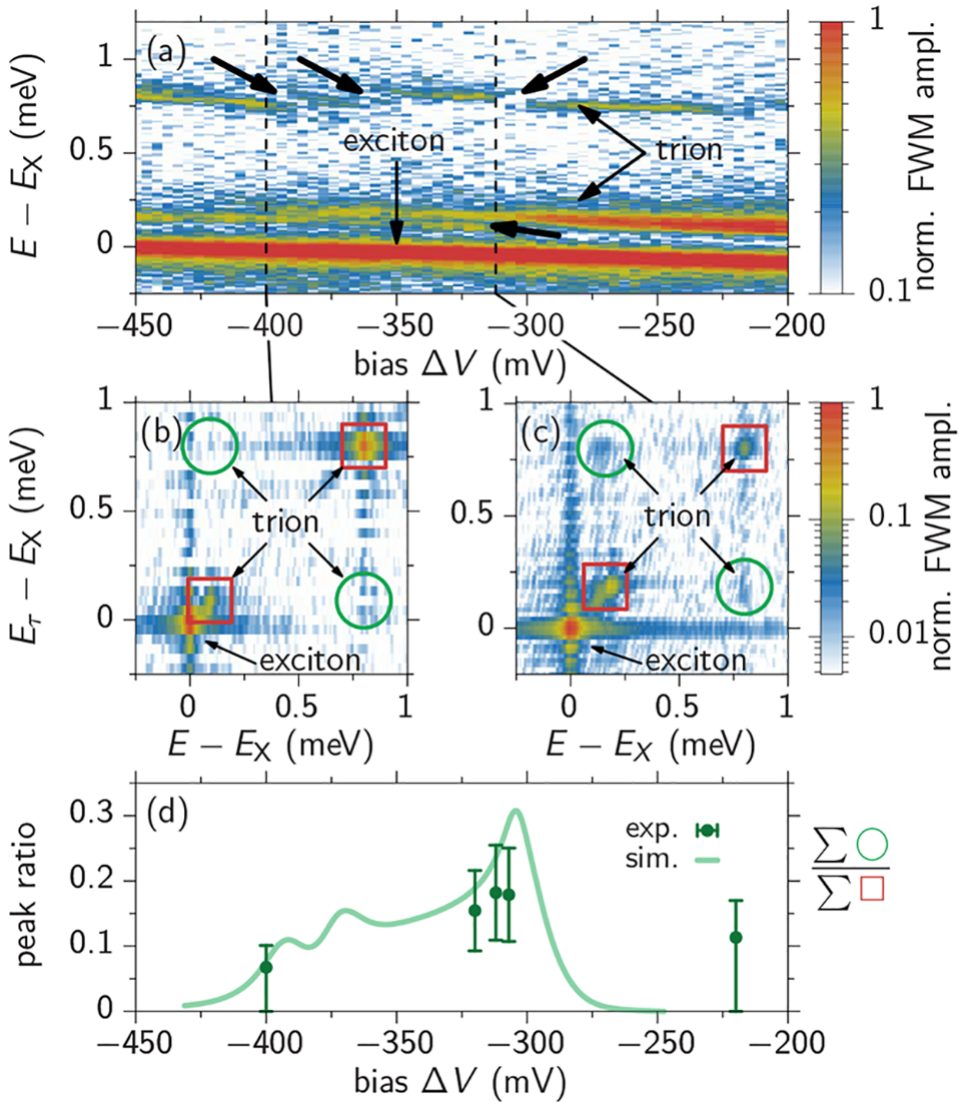
(a) Measured bias scan of the QDM FWM spectra with pulse delays τ_12_ = τ_23_ = 0.1 ps. The arrows correspond to those in Figure 3b. (b, c) Exemplary 2D FWM spectra at the bias values marked in (a). Red boxes mark the diagonal peaks and green circles the off-diagonal peaks used to determine the peak ratios in (c). (d) Peak ratios in the 2D FWM spectra to quantify the coherent coupling as a function of applied bias. Experiment as dots and simulation as line with homogeneous and inhomogeneous dephasing rates γ = 0.05 meV and σ = 0.15 meV, respectively.

In Figure 4a we show the respective measured bias scan of the FWM spectra for small pulse delays τ_12_ = τ_23_ = 0.1 ps which maximize the FWM response and can still be treated as τ_12_ = τ_23_ = 0 in the simulation. Note that the depicted bias range differs from the PL measurement in Figure 2a. The reason is that the state energies strongly depend on the optical excitation conditions (see SI for details). Therefore, the bias ranges in PL and FWM cannot be compared directly. The lowest energetic line stems from a neutral exciton. Therefore, it involves different charge states of the system and does not interact with the trion transition lines. We thus use it to phase-correct the delay scans, to generate 2D FWM spectra (see Figure 4b,c and SI). The upper two spectral lines clearly belong to the trion transitions we are interested in. Obviously, much fewer spectral features are visible compared to the measured PL spectra (Figure 2a), which is again related to the fact that the FWM signal is nonlinear and stems from the χ^(3)^ response of the optical transitions. Therefore, one naturally expects spectral lines that are weaker in PL to be strongly suppressed in FWM. Consequently, they might drop below the signal-to-noise level and do not appear in the detected spectra. Nevertheless, by comparing the measurement in Figure 4a with the simulation in Figure 3 we find striking similarities. First of all, we observe the same intensity distribution with stronger low-energy lines at larger electric fields (Δ*V* ≈ −200 mV) and stronger high-energy lines at smaller electric fields (Δ*V* ≈ −450 mV). We also see one small indication of an avoided crossing in the low-energy line at Δ*V* ≈ −300 mV (upward left-pointing arrow), while no such feature appears in this spectral line at smaller fields. We further find a few indications of avoided crossings in the transition line at higher energies. Although only slightly above the noise-level, we can identify three gaps in the spectral line, namely at Δ*V* = −390, −360 (downward right-pointing arrows), and −300 mV (downward left-pointing arrow). These observations are in agreement with the predictions from the model in Figure 3. Our ***k***·***p*** model with an extended electron and hole state basis and a QDM morphology mapped, as far as possible, from a typical grown QDM structure yields FWM spectra that exhibit exactly the same features. It shows two horizontal branches interrupted by avoided crossings, and their intensities decay in the opposite bias directions and no additional transitions are visible (SI). This validates the applicability of the semiempirical model, but may open the path to study the effect of composition and morphology on the nonlinear optical response and quantum coherence properties of the QDM.

We move forward to the main result, which is the demonstration and control of the quantum coherence transfer between different trion complexes in the QDM. The bias-controlled coherent coupling of the QDM is revealed by measuring 2D FWM spectra, as exemplarily depicted in Figure 4b,c. The two energy axes of these 2D spectra are on the one hand determined by the spectrometer in the emitted signal (horizontal, *E*) and on the other hand by a Fourier transform with respect to the delay τ_12_ in the FWM measurement (vertical, *E*_τ_). The meaning of the different peaks in the maps can directly be read from the plots: Optical absorption and FWM emission from the same trion is represented by the peaks on the diagonal of the plot (red squares). Off-diagonal peaks, at the positions marked by the green circles, stem from an optical absorption from one trion (*E*_τ_ axis) and FWM generation from another one (*E* axis), due to their coherent tunnel-coupling. Therefore, the presence of these peaks in Figure 4c confirms the coherent quantum state transfer for the electric field, where the two hole/ground states are in resonance (Δ*V* ≈ −300 mV). We see that the off-diagonal peaks are suppressed in Figure 4b (Δ*V* = −400 mV), indicating a much less efficient coherence transfer for electric fields where the excited states are not in close resonance.

Despite the limited signal-to-noise ratio of the 2D spectra, which makes a consistent quantitative analysis challenging, we carefully determine the relative strength of the off-diagonal peaks with respect to the diagonal ones, and therefore quantify the coherent coupling between the QDs. Special attention has to be payed to disregard the impact of the dominant neutral exciton peak, marked in the plots (SI). We then determine the peak visibility by calculating the ratio of the sum-intensity of the off-diagonal peaks with respect to the diagonal ones, as symbolically written next to Figure 4d. In this figure, the peak ratios from the performed experiments are shown as green dots, and the corresponding simulation as pale green curve. For the simulation we consider a similar spectral broadening as in Figure 3 (SI). Both, experiment and theory show increased peak ratios around the avoided crossing at a bias of Δ*V* ≈ – 300 mV and the measurement and the simulation show a reasonable agreement. Note that to match the experiment we have to assume additional inhomogeneous dephasing due to fluctuations of the transition energies with a standard deviation of σ = 0.15 meV (see SI for details) .^46^ In short, an additional dephasing of the cross-coherences , exceeding the pure dephasing acting on the optical active coherences , will reduce the visibility of the off-diagonal peaks in the 2D spectrum. Such an additional dephasing is expected for coherences between states differing in charge distribution due to their large sensitivity to environmental fluctuations.

### Minimum Model Interpretation

**Figure 5:**
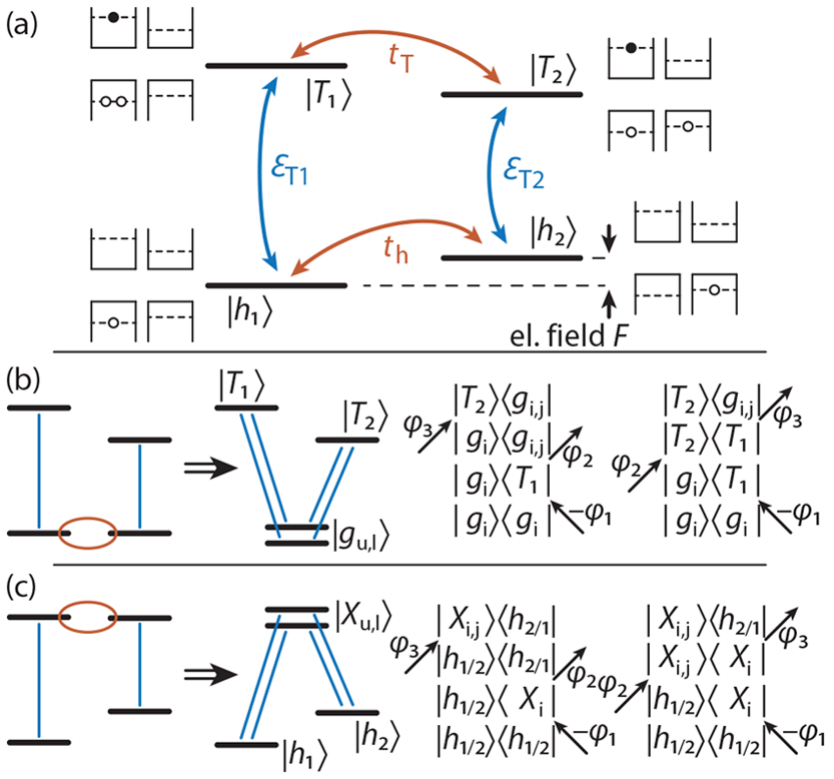
(a) Schematic picture of the minimum model to explain the coherent coupling effect. We include two hole  and two trion states  in the two QDs as depicted next to each level. We consider an optical transitions  (blue) and tunnel couplings between the hole and the trion states (orange). (b, c) Illustration of the hole (b) and trion resonances (c). Left: Exciton level structure with optical transitions (blue lines) and tunnel coupling (orange ellipses). Middle: New eigenstates forming ground  (b) and excited states  (c) doublets of upper (u) and lower (l) states. Right: Relevant double-sided Feynman diagrams leading to the off-diagonal (coherent coupling) peaks in the 2D spectra with i, j = u, l.

To fully understand the transfer of the coherence between the trions in the FWM process, we developed a minimum model that only considers one ground hole state per QD  and the corresponding bright trion states with an additional electron–hole pair in one of the dots , which makes a total of four levels similar to refs (25) and (27). A schematic of the coupling mechanisms between these states is depicted in Figure 5a. While optical excitations  are only possible within each trion complex (T1, T2, vertical connection), the tunnelings *t*_h,T_ connect the hole (h) and trion (T) states (horizontal connection), respectively. This system can be described by the Hamiltonian:
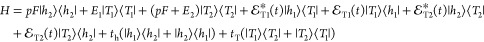
When we now assume that the two hole states are in resonance we can neglect the tunneling between the trion states, because they are far from resonance (*t*_T_ = 0). This situation is schematically shown in Figure 5b, left, where the tunnel coupling between the hole states leads to a hybridization resulting in a new upper  and lower ground state  (middle). The optical active transitions now connect both of these states to each trion state resulting in a V-level structure. To construct the FWM signals with the phase combination φ_3_ + φ_2_ – φ_1_, we consider the two double-sided Feynman diagrams on the right. We see that the V-level structure induced by the hole tunnelling results in different possibilities to have an absorption in  and an emission in  (the other off-diagonal peak is retrieved by exchanging 1 and 2). The first diagram reaches the coherence transfer between the two trion complexes via the ground state doublet  after φ_2_ and the second one via the intertrion coherence . It is interesting to note that coherent coupling is mediated via the common ground states where the hole states are delocalized across the two dots, but in the trion states the hole states are localized in each dot. The lifted degeneracy of the ground states in theory leads to the generation of multiple peaks on the off-diagonal, which cannot be resolved in the experiment but are visible in the simulated 2D spectra in the SI. An equivalent situation is found when the trion states are in resonance forming new excited states  resulting in an Λ-level scheme, as depicted in Figure 5c. Here, the initial state is a thermal mixture of the hole states  and  and the Feynman diagrams show that the path of coherence transfer goes via the hole states  or the excited states doublet  to the respective other hole state.

Before the FWM pulse sequence is started, the QDM is typically in its ground states, i.e., in a thermal equilibrium of the hole states. However, recently an experimental scheme has been developed to deterministically initialize the QDM in one of the two hole states.^49^ It is thus becoming possible to induce a unidirectional coherence transfer between the two QDs. This would manifest in the 2D FWM spectrum with the appearance of only one off-diagonal peak. One would be starting from a noneigenstate of the system which introduces additional dynamics in the system. It is important to note that this FWM scheme offers the possibility to probe different coherence dynamics that are not optically active, namely , , , and . To achieve this, one would have to vary the delay between the second and third pulse (τ_23_), as can be seen from the Feynman diagrams in Figure 5b,c.

## Conclusions

In summary, revealing controlled coherent coupling between different trion complexes in a QDM renders a major advance in the field of photonic quantum technology. Our proof-of-principle demonstration of nonlinear multiwave mixing spectroscopy on these systems opens the door to address coherences that are not optically active, making them significantly longer lived than their bright counter parts and could therefore be used as quantum storage.^25,27^ To improve the performance of quantum devices, current work aims to generate singlet–triplet qubits,^50−52^ which are expected to be robust against decoherence from electrostatic and magnetic fluctuations in the solid. Due to its selective access to different quantum coherences, the FWM method stands out as an excellent tool to exploit this new benchmark for quantum computing with solid state qubits.

## Supporting Information Available

S1, Four-wave mixing experiment; S1A, Experimental method and sample; S1B, Coherence dynamics; S1C, Rabi rotations; S1D, Identification of the neutral exciton-biexciton complex; S1E, Power dependence of PL spectra; S1F, Fitting 2D FWM spectra; S2, Theory; S2A, Semiempirical model; S2B, Calculation of 2D spectra for ultrashort pulses; S2C, Dynamical simulations; S2D, Inhomogeneous dephasing; S2E, ***k***·***p*** model graphical TOC entry (PDF)
