# Supplementary material for: Controlled Coherent Coupling in a Quantum Dot Molecule Revealed by Ultrafast Four-Wave Mixing Spectroscopy
Source: ACS Photonics. 2023 May 8;10(5):1504–11. doi: 10.1021/acsphotonics.3c00108 (PMC10197170; doi:10.1021/acsphotonics.3c00108)
Supplement: Supplementary file 1 — ph3c00108_si_001.pdf [file ph3c00108_si_001.pdf]

Controlled Coherent Coupling in a Quantum Dot Molecule Revealed by Ultrafast Four-Wave Mixing Spectroscopy (*Supporting Information*)

Daniel Wigger,[*,†,‡] Johannes Schall,[¶] Marielle Deconinck,[¶] Nikolai Bart,[§] Paweł Mrowiński,[†,‖] Mateusz Krzykowski,[†] Krzysztof Gawarecki,[†] Martin von Helversen,[¶] Ronny Schmidt,[¶] Lucas Bremer,[¶] Frederik Bopp,[⊥] Dirk Reuter,[#] Andreas D. Wieck,[§] Sven Rodt,[¶] Julien Renard,[@] Gilles Nogues,[@] Arne Ludwig,[§] Paweł Machnikowski,[†] Jonathan J. Finley,[⊥] Stephan Reitzenstein,[¶] and Jacek Kasprzak[*,⊥,@]

†*Institute of Theoretical Physics, Wrocław University of Science and Technology, 50-370 Wrocław, Poland*

‡*School of Physics, Trinity College Dublin, Dublin 2, D02 PN40, Ireland*

¶*Institute of Solid State Physics, Technische Universität Berlin, 10623 Berlin, Germany*

§*Lehrstuhl für Angewandte Festkörperphysik Ruhr-Universität Bochum, 44780 Bochum, Germany*

‖*Laboratory for Optical Spectroscopy of Nanostructures, Department of Experimental Physics, Wrocław University of Technology, 50-370 Wrocław, Poland*

⊥*Walter Schottky Institut and Physik Department, Technische Universität München, 85748 Garching, Germany*

#*Department Physik, Universität Paderborn, 33098 Paderborn, Germany*

@*Université Grenoble Alpes, CNRS, Grenoble INP, Institut Néel, 38000 Grenoble, France*

E-mail: daniel.wigger@tcd.ie; jacek.kasprzak@neel.cnrs.fr

This document contains:

S1. Four-wave mixing experiment
- **S1A. Experimental method and device**
- **S1B. Coherence dynamics**
- **S1C. Rabi rotations**
- **S1D. Identification of the neutral exciton-biexciton complex**
- **S1E. Power dependence of PL spectra**
- **S1F. Fitting 2D FWM spectra**

S2. Theory
- **S2A. Semi-empirical model**
- **S2B. Calculation of 2D spectra for ultra-short pulses**
- **S2C. Dynamical simulations**
- **S2D. Inhomogeneous dephasing**
- **S2E. $\boldsymbol{k}\cdot\boldsymbol{p}$ model**

S1. Four-wave mixing experiment

In this section we present details on the performed experiment and used sample (Sec. S1A). We show standard characterization measurements of the coherence (Sec. S1B) and light-matter coupling (Sec. S1C). Further, we identify the neutral exciton transition (Sec. S1D), demonstrate the power dependence of the photoluminescence (PL) spectra (Sec. S2E), and describe our fitting procedure for the 2D spectra (Sec. S1F).

S1A. Experimental method and device

We employ the heterodyne spectral interferometry technique introduced in Ref.[1] to detect four-wave mixing (FWM) signals from the quantum dot molecule (QDM). Its two-beam version was described in detail in the Supplementary Material of Ref.[2], while its extension to a three-beams configuration was presented in the Supplementary Material of Ref.[3]. We use a Ti:Sapphire femto-second oscillator tuned into resonance with bright excitonic (trion) transitions of the QDM around 930 nm (1333 meV). In order to avoid driving spectrally nearby transitions, for instance biexcitons, the pulses are spectrally shaped (with a passive, diffraction grating-based pulse shaper) increasing their duration to ≈ 0.4 ps (full width at half maximum of the intensity spectrum). The main beam is then split into the reference pulse $\mathcal{E}_{\mathrm{R}}$ and three excitation pulses $\mathcal{E}_{1,2,3}$. In the latter, the consecutive pulses in the train are distinctly phase-shifted using acousto-optic modulators driven at the radio-frequencies

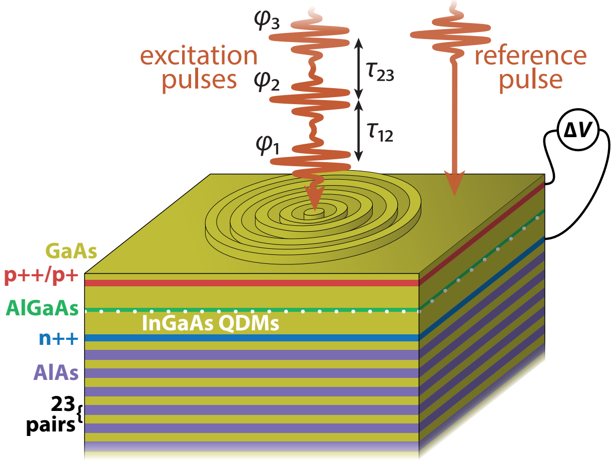

Figure S1: Schematic picture of the sample structure. Above a GaAs/AlAs distributed Bragg structure the InGaAs quantum dot molecule (QDM) layer is sandwiched between charge-doped layers, which are connected to a bias source. For details on the sample design and processing we refer to Ref.[5]. The optical in- and out-coupling is improved by a circular Bragg grating, also called a bulls-eye photonic structure, on the top surface. The laser pulses used for the optical excitation in the four-wave mixing experiment are focused on the center of the grating, while the reference beam hits the free surface in the vicinity.

$(\Omega_1, \Omega_2, \Omega_3) = (80, 79, 79.77)$ MHz. Using mechanical delay stages, they then acquire respective delays τ_{12} and τ_{23}, between the first two and last two arriving beams, respectively. $\mathcal{E}_{1,2,3}$ are recombined into the same spatial mode and propagate unidirectionally. Using an external microscope objective, $\mathcal{E}_{1,2,3}$ are focused on the sample surface, impinging the central part of the circular Bragg grating, while $\mathcal{E}_{\mathrm{R}}$ is placed on the flat surface around 15 µm apart (Fig. S1). The signal is collected in the reflection direction. The reflection from the QDM, which also contains the FWM signal, is interfered with $\mathcal{E}_{\mathrm{R}}$ and heterodyned at the frequency $\Omega_3 + \Omega_1 - \Omega_1$ in the same manner as explained in previous publications.[1–4] Note, that in this work we apply the degenerate two-pulse FWM scheme, i.e., $\tau_{23} = 0$.

The sample is kept in a He-flow cold-finger cryostat at the temperature of 7 K. To tune the spectral separation between quantum levels in the QDM, we vary the gate voltage ΔV (Fig. S1), supplied by an external voltage source micro-bonded to the electrodes of the QDM device via the feed-through connectors of the cryostat. In the initial part of the experiment, the current-voltage characterization was carried out (not shown). To assure a safe operation of the p-i-n diode the current is limited to 80 µA. The voltage source, provided by *Keithley*, is interfaced via the General Purpose Interface Bus (GPIB) with the home-developed software controlling the whole experiment.

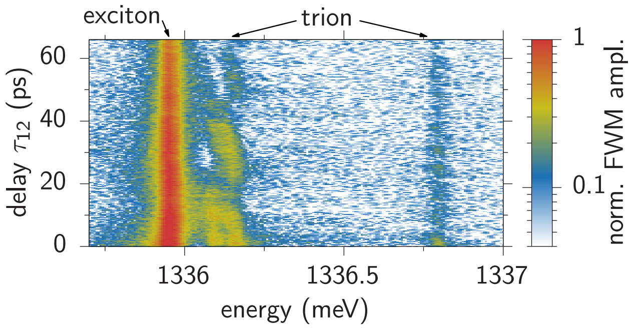

Figure S2: Spectral dynamics of the trion transitions as a function of the delay τ_{12}. The trion lines show a coherence beating due to the coherent coupling. The exciton line just decays due to the decoherence of the zero phonon line.

S1B. Coherence dynamics

Figure S2 shows an exemplary coherence dynamics measurement by varying the delay τ_{12} in the FWM experiment. While the neutral exciton line only decays due to dephasing processes, the trion transitions exhibit pronounced oscillations. These involved dynamics originate from the coherent coupling between the different states and lead to the off-diagonal peaks in the 2D FWM spectra (see e.g. Fig. S7).

The beat dynamics in the FWM signal is suppressed when only one of the transitions is optically driven. For such a measurement the spectrally integrated FWM amplitude of the neutral exciton is shown in Fig. S3. By fitting the long-time decay with a single exponential function, we deduce a dephasing time of 200 ps. This dephasing rate is used for the simulations in Fig. S10. The initial rapid drop of the FWM amplitude is most likely stemming from a phonon-induced dephasing process[6] and is not further investigated here.

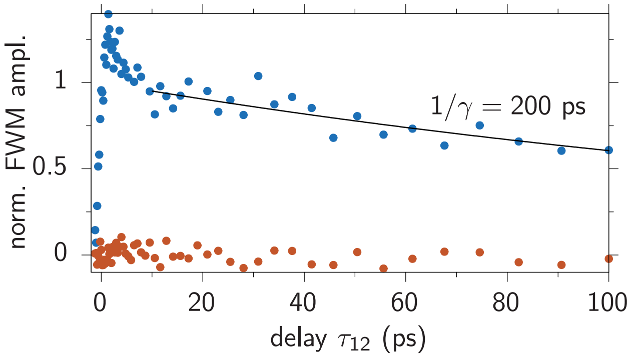

Figure S3: Spectrally integrated coherence dynamics as a function of τ_{12} of the neutral exciton line, measured far from the hole tunneling resonance (blue points). The orange points represent the noise level. The long-time decay is fitted with a single exponential with the dephasing time 200 ps. The initial faster decay is due to phonon-induced dephasing.

S1C. Rabi rotations

To characterize the strength of the light-matter coupling of the QDM in Fig. S4 we plot the spectrally integrated FWM amplitude (for $\tau_{12} = 0$) as a function of the applied laser pulse amplitude. We find the expected Rabi rotation behavior,[7] which shows that a pulse area of $\pi/2$ is found for a pulse amplitude around $\sqrt{P_1} = 0.7\,\sqrt{\mu\mathrm{W}}$. For all measurement in this work we restrict ourselves to small excitation powers in the linear regime of the Rabi rotation, i.e., $\sqrt{P_1} < 0.05\,\sqrt{\mu\mathrm{W}}$.

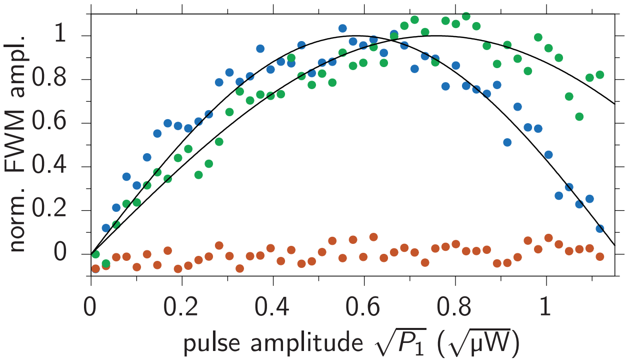

Figure S4: Rabi rotation experiment, performed on the neutral exciton (blue points) and the trion (green points) transitions. The orange points represent the noise level. Pulse areas of $\pi/2$ are found for pulse amplitudes around $0.7\,\sqrt{\mu\mathrm{W}}$.

S1D. Identification of the neutral exciton-biexciton complex

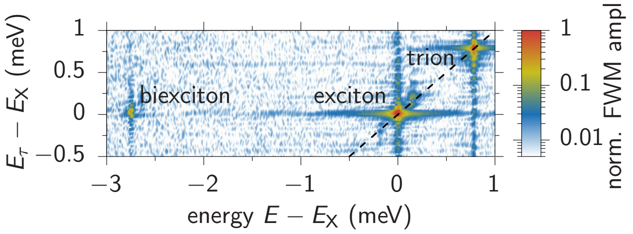

Figure S5: 2D FWM spectrum showing the exciton-biexciton transition as off-diagonal peak. The exciton and trion transitions appear on the diagonal.

As stated in the main text, the bright spectral line in Fig. 3(a) stems from a neutral exciton transition. The standard proof of this type of transition is the detection of a coupled biexciton state.[2] We do this through the 2D FWM spectrum in Fig. S5, where the biexciton

appears as off-diagonal peak with a binding energy of around 2.8 meV, which is a typical value for InGaAs QDs.[8]

S1E. Power dependence of PL spectra

We have seen in the main text, that the bias ranges where the hole and trion resonances appear differ between the presented PL and FWM measurements. In Fig. S6(a) we show the same data as in Fig. 1(a) in the main text for an optical excitation power of $P = 100$ nW and in (b) the same measurement for $P = 400$ nW. We find the same spectral features of line-shifts and avoided-crossings but on different bias intervals. The range is not only shifted but also scaled by almost a factor of 2. This shows that the bias-dependence of the different state energies strongly depends on the specific optical excitation conditions. We also note that even more different scenarios are observed upon additional white light illumination (not shown). Varying optical excitation, changes the free carrier density and thus also the energetic level structure in the p-i-n diode and also the alignment of the quantized levels in the QDM itself. Therefore, because already the general optical excitation schemes in PL and FWM are off-resonant and resonant, respectively, and consequently entirely different, the two experiments cannot be quantitatively compared regarding their bias range properly.

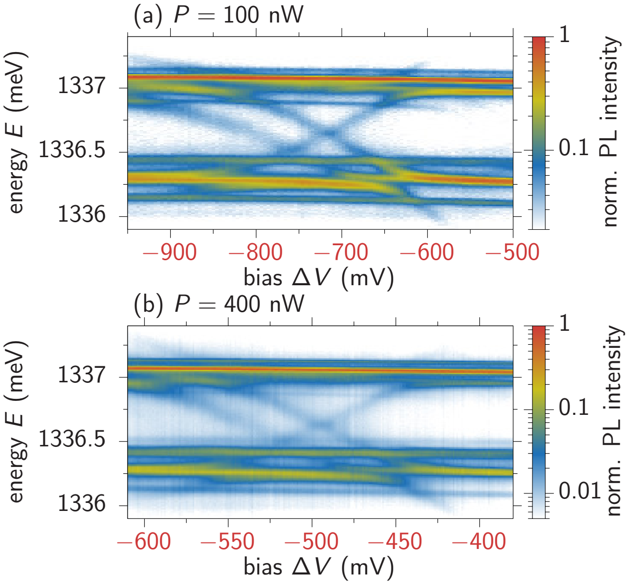

Figure S6: Bias scans of the PL intensity for two different excitation powers. (a) Same as Fig. 1(a) in the main text with $P = 100$ nW. (b) 400 nW depicted for a different bias range than (a).

S1F. Fitting 2D FWM spectra

To efficiently remove the prominent impact of the neutral exciton transition from the 2D spectra depicted in Fig. S7 we fit each spectrum with a sum of 2D Lorentzians. While for most studied bias values we use one diagonal peak for the exciton and two diagonal and two off-diagonal ones for the trion transitions, for the bias values $\Delta V = -307$, -312, and -320 mV we have to consider one additional diagonal and two additional off-diagonal peaks. This appearance of additional peaks in the bias range directly reflects the development of an avoided-crossing in the linear spectra (see Fig. 3(a) in the main text).

Our goal is to determine the peak ratio in the 2D spectra consistently in the measured and the simulated data. Therefore, we simply focus on four parts marked by colored areas (dashed squares) in Fig. S7(o). The signal amplitude summed over the red shaded areas gives the diagonal contributions and the sum over the green shaded areas the off-diagonal ones. This procedure can be easily automated to retrieve the peak ratios (green areas/red areas) for many bias values in the simulation, i.e., many 2D spectra. To determine the experimental peak ratios we use the fitted 2D spectra to reduce the uncertainties stemming from different discretization or spectral oscillations due to insufficiently long delay ranges.

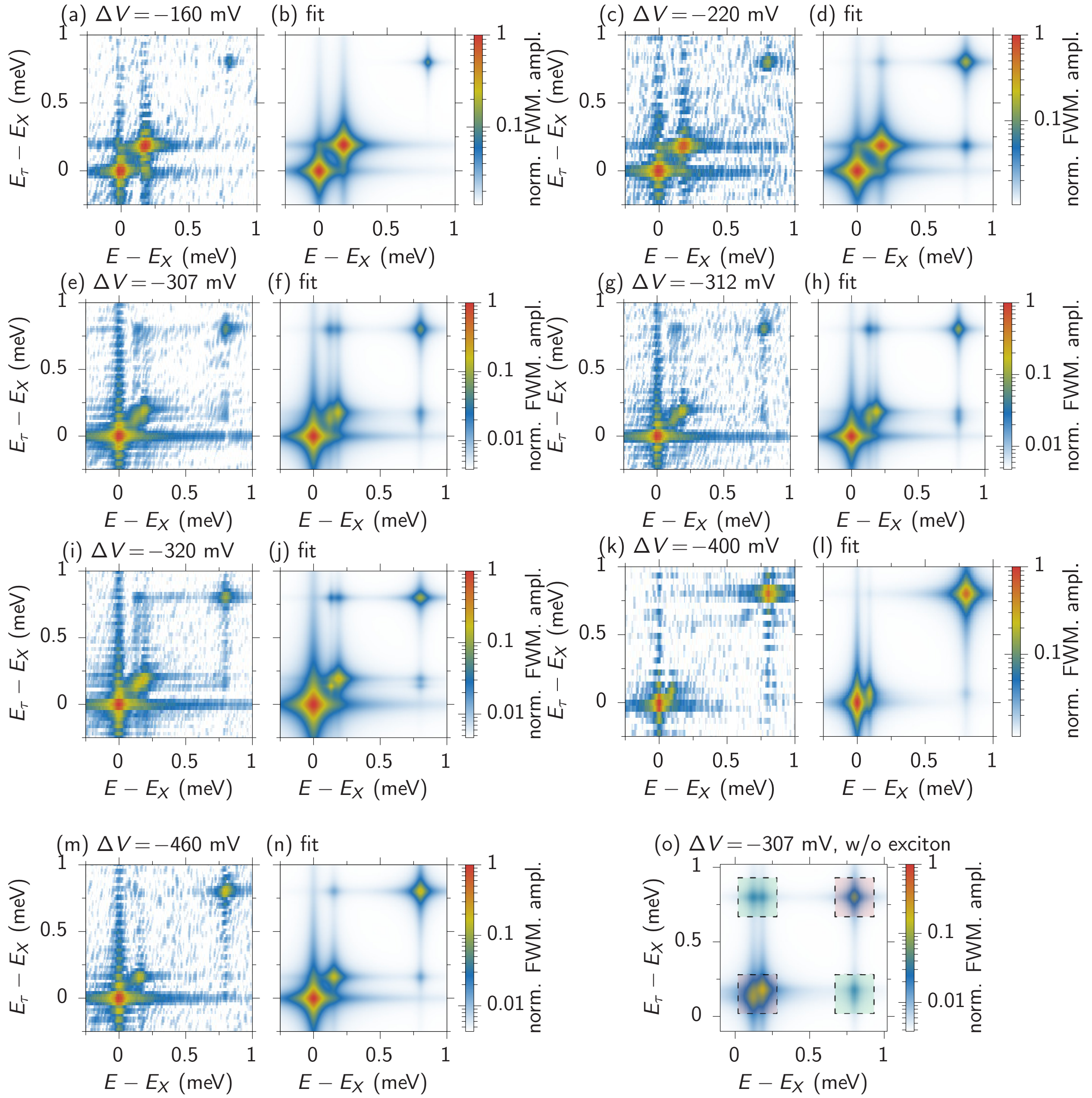

Figure S7: (a)-(n) Measured 2D FWM spectra with respective fits for different bias values. The fit function is a sum of 2D Lorentzians. (o) Fitted 2D FWM spectrum for $\Delta V = -307$ mV without the neutral exciton line. The red shaded areas mark the integration range for the diagonal contributions, the green areas those for the off-diagonal ones.

S2. Theory

Here, we present the details of the semi-empirical theory for the coupled hole and trion system (Sec. S2A). This model yields transition energies and amplitudes for dipole-allowed transitions that are used for determining the FWM spectrum from the analytical formulas in

the ultra-short pulse limit (Sec. S2B) or from dynamical simulations (Sec. S2C). Further, we discuss the influence of an inhomogeneous dephasing on the 2D spectra (Sec. S2D). Finally, we present the $\boldsymbol{k}\cdot\boldsymbol{p}$ model and the spectra calculated from the system's eigenstates and eigenenergies obtained in this way (Sec. S2E).

S2A. Semi-empirical model

In the following electron and hole destruction (creation) operators are given by $a_\sigma^{(\dagger)}$ and $h_{n,\sigma}^{(\dagger)}$ for the spin orientation $\sigma \in \{\uparrow,\downarrow\}$, respectively, and $n \in \{1,2\}$ indicates in which QD the hole is located.

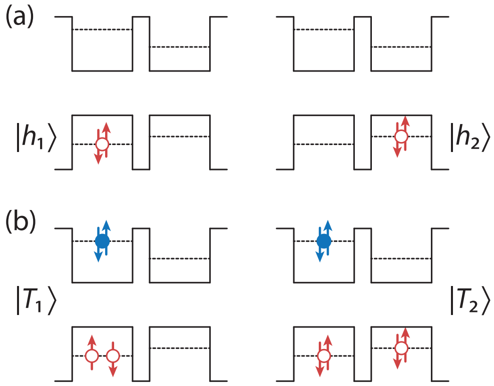

Figure S8: Schematic picture illustrating the different (a) hole and (b) trion states in the QDM taking into account the spins of the particles.

The relevant single particle states of the QDM are given by the single hole states forming the ground states, which are characterized by the QD number and the spin orientation: $|1_\uparrow\rangle, |1_\downarrow\rangle, |2_\uparrow\rangle, |2_\downarrow\rangle$. The relevant trions forming the excited states emerge by the creation of an additional electron-hole pair, with the electron always in QD1. These three-particle complexes now possess two holes, whose spin coupling results in new eigenstates that can be

sorted into singlet $\left|S_{(m,n)}\right\rangle$ $(m, n \in \{1,2\})$ and triplet states $|T_\alpha\rangle$ $(\alpha \in \{+,-,0\})$ defined by

$$\left|S_{(2,0)}\right\rangle = h^\dagger_{1,\uparrow} h^\dagger_{1,\downarrow} \left|0\right\rangle \tag{S1a}$$

$$\left|S_{(1,1)}\right\rangle = \frac{1}{\sqrt{2}} \left(h^\dagger_{1,\uparrow} h^\dagger_{2,\downarrow} - h^\dagger_{1,\downarrow} h^\dagger_{2,\uparrow} \right) \left|0\right\rangle \tag{S1b}$$

$$\left|S_{(0,2)}\right\rangle = h^\dagger_{2,\uparrow} h^\dagger_{2,\downarrow} \left|0\right\rangle \tag{S1c}$$

$$\left|T_{+}\right\rangle = h^\dagger_{1,\uparrow} h^\dagger_{2,\uparrow} \left|0\right\rangle \tag{S1d}$$

$$\left|T_{-}\right\rangle = h^\dagger_{1,\downarrow} h^\dagger_{2,\downarrow} \left|0\right\rangle \tag{S1e}$$

$$\left|T_{0}\right\rangle = \frac{1}{\sqrt{2}} \left(h^\dagger_{1,\uparrow} h^\dagger_{2,\downarrow} + h^\dagger_{1,\downarrow} h^\dagger_{2,\uparrow} \right) \left|0\right\rangle \tag{S1f}$$

To get the full trion wave function we have to add the spin orientation $\sigma \in \{\uparrow, \downarrow\}$ of the remaining electron to the states such that we get $\left|\sigma, S_{(m,n)}\right\rangle$ and $|\sigma, T_\alpha\rangle$. Overall we have 4 ground/hole states and 12 excited/trion states.

For the minimal model introduced in the main text we may identify $|h_1\rangle \to |1_\uparrow\rangle$, $|h_2\rangle \to |2_\uparrow\rangle$, $|T_1\rangle \to |S_{2,0}\rangle$, and $|T_2\rangle \to |S_{1,1}\rangle$ (see Fig. S8).

The ground/hole state Hamiltonian reads:

$$H_{\mathrm{h},\sigma} = \sum_{\sigma=\uparrow,\downarrow} \left[(V_2 - F) \left|2_\sigma\right\rangle\!\left\langle 2_\sigma\right| + t \left(\left|1_\sigma\right\rangle\!\left\langle 2_\sigma\right| + \mathrm{h.c.} \right) \right], \tag{S2}$$

where V_2 is the energy difference between the ground states in the two QDs, $F = ed\mathcal{E}_z$ is the dipole energy associated with the charge displacement in the axial electric field $\mathcal{E}_z$, where d is the effective distance between the QDs and e is the elementary charge, and t is the tunnel coupling.

The excited/trion state Hamiltonian reads (dropping the conserved electron spin index σ on the right-hand side):

$$\begin{aligned} H_{\mathrm{T},\sigma} = J\Bigg(& \left|S_{(1,1)}\right\rangle\!\left\langle S_{(1,1)}\right| - \sum_{+,-,0} \left|T_\alpha\right\rangle\!\left\langle T_\alpha\right| \Bigg) \\ & + 2V_\mathrm{c} \left|S_{(0,2)}\right\rangle\!\left\langle S_{(0,2)}\right| \\ & + \sum_{m,n=1,2} n \left(V_2' - F\right) \left|S_{(m,n)}\right\rangle\!\left\langle S_{(m,n)}\right| \\ & + \sum_{\alpha=+,-,0} \left(V_2' - F\right) \left|T_\alpha\right\rangle\!\left\langle T_\alpha\right| \\ & + t \left(\left|S_{(1,1)}\right\rangle\!\left\langle S_{(0,2)}\right| + \left|S_{(1,1)}\right\rangle\!\left\langle S_{0,2}\right| + \mathrm{h.c.} \right). \end{aligned} \tag{S3}$$

Here, J is the hole exchange splitting, V_c is the Coulomb energy of exciton dissociation, and

V_2' is the hole energy difference between the QDs in the presence of the exciton (taking the Coulomb correlation energy into account). The total trion Hamiltonian is

$$H_{\mathrm{T}} = H_{\mathrm{T},\uparrow} + H_{\mathrm{T},\downarrow} \tag{S4}$$

The Hamiltonian for the electron-hole short-range exchange interaction is most conveniently written in the second quantization form, reading

$$H^{\mathrm{ex}} = \delta \left(a_{\uparrow}^{\dagger} h_{\downarrow}^{\dagger} h_{\downarrow} a_{\uparrow} + a_{\downarrow}^{\dagger} h_{\uparrow}^{\dagger} h_{\uparrow} a_{\downarrow} \right) . \tag{S5}$$

The considered values of the system parameters are $V_{\mathrm{c}} = 20$ meV, $V_2 = 0$ (this parameter merely sets the reference for the electric field; with this choice the $F = 0$ is fixed at the hole resonance), $V_2' = 0.785$ meV, $\delta = 0.21$ meV, $J = 0$. With these parameters, the model is equivalent to the one used in Ref.[5]

The dipole operators for σ_+ and σ_- polarized light are, respectively,

$$M^{(+)} = h_{1\uparrow} a_{\downarrow} = |1_{\downarrow}\rangle\langle\downarrow, S_{(2,0)}| + |2_{\downarrow}\rangle \left(\frac{1}{\sqrt{2}} \langle\downarrow, S_{(1,1)}| + \langle\downarrow, T_-| + \frac{1}{\sqrt{2}} \langle\downarrow, T_0| \right) , \tag{S6}$$

and

$$M^{(-)} = h_{1\downarrow} a_{\uparrow} = |1_{\uparrow}\rangle\langle\uparrow, S_{(2,0)}| + |2_{\uparrow}\rangle \left(\frac{1}{\sqrt{2}} \langle\uparrow, S_{(1,1)}| + \langle\uparrow, T_+| + \frac{1}{\sqrt{2}} \langle\uparrow, T_0| \right) , \tag{S7}$$

which determines the optically active transitions in the system.

S2B. Calculation of 2D spectra for ultra-short pulses

Using the matrix of transition amplitudes between the hole and trion states M_{iu}, we can derive the two-pulse FWM spectrum in a general analytical way in the limit of ultra-short laser pulses.

The diagonalized Hamiltonian is of the form

$$H = \sum_i E_i |\mathfrak{h}_i\rangle\langle\mathfrak{h}_i| + \sum_u E_u |\mathfrak{T}_u\rangle\langle\mathfrak{T}_u| + [\mathcal{E}(t+\tau_{12})e^{i\varphi_1} + \mathcal{E}(t)e^{i\varphi_2} + \mathrm{h.c.}]M , \tag{S8}$$

where $|\mathfrak{h}_i\rangle$ and $|\mathfrak{T}_j\rangle$ are the hole and electron eigenstates, respectively, with the corresponding eigenenergies E_i and E_u,

$$\mathcal{E}(t) = \hat{\mathcal{E}}(t)e^{-i\omega t},$$

and M is one of the operators in Eq. (S6) or Eq. (S7), depending on the polarization of light. In the interaction picture this reads

$$\tilde{H}(t) = [\mathcal{E}(t+\tau_{12})e^{i\varphi_1} + 2\mathcal{E}(t)e^{i(\varphi_2+\varphi_3)} + \text{h.c.}]\tilde{M}(t)\,,$$

where

$$\tilde{M}(t) = \sum_{i,u} M_{iu}\,|\mathfrak{T}_u\rangle\langle\mathfrak{h}_i|\,e^{i\omega_{iu}t}\,,$$

with $\omega_{iu} = (E_u - E_i)/\hbar$.

Before any optical excitation the system state is a mixture of hole states represented by a density matrix $\rho^{(0)}$. The first optical excitation happens at $t = -\tau_{12}$ and we assume that the laser pulse is much faster than any internal dynamics, i.e., dephasing, tunneling, or decay. The density matrix immediately after this pulse is then given by

$$\begin{aligned}\rho^{(1)} &= -\frac{i}{\hbar}\int\limits_{-\tau_{12}-\varepsilon}^{-\tau_{12}+\varepsilon}\left[\tilde{H}(t),\rho^{(0)}\right]\,\mathrm{d}t\\ &= -\frac{i}{\hbar}\int\limits_{-\infty}^{\infty}\hat{\mathcal{E}}^*(t+\tau_{12})e^{-i\varphi_1}\rho^{(0)}\sum_{i,u}M^*_{iu}\,|\mathfrak{h}_i\rangle\langle\mathfrak{T}_u|\,e^{-i\omega_{iu}t}e^{-i\omega(t+\tau_{12})}\,\mathrm{d}t + \text{h.c.}\,,\end{aligned} \tag{S9}$$

where h.c. contains the phase $+\varphi_1$, which is irrelevant for the final FWM signal and can therefore directly be neglected. After introducing the pulse spectrum,

$$\hat{s}(\omega) = \int\limits_{-\infty}^{\infty}\hat{\mathcal{E}}(t)e^{-i\omega t}\,\mathrm{d}t, \tag{S10}$$

the FWM-relevant density matrix after the first pulse reads

$$\rho^{(1)}(\tau_{12}) = -\frac{i}{\hbar}e^{-i\varphi_1}\rho^{(0)}e^{i\omega_{iu}\tau_{12}}\sum_{i,u}M^*_{iu}\,|\mathfrak{h}_i\rangle\langle\mathfrak{T}_u|\,\hat{s}^*(\omega-\omega_{iu}) = \sum_{iu}\rho^{(1)}_{iu}\,|\mathfrak{h}_i\rangle\langle\mathfrak{T}_u|\,. \tag{S11}$$

Before the interaction with the second pulse, the system can undergo dephasing and decay dynamics, such that the density matrix immediately before the next pulse is given by

$$\rho^{(1)}(0-\varepsilon) = \sum_{i,u}\rho^{(1)}_{iu}(\tau_{12},\omega)\,|\mathfrak{h}_i\rangle\langle\mathfrak{T}_u|\,e^{-\gamma_{iu}\tau_{12}}\,, \tag{S12}$$

where γ_{iu} is the dephasing rate for a given coherence.

The second pulse acts via a second order process, which leads to the density matrix

$$\rho^{(2)} = -\frac{1}{\hbar^2}\int\limits_{-\varepsilon}^{\varepsilon}\int\limits_{-\varepsilon}^{t}\left[\tilde{H}(t),\left[\tilde{H}(t'),\rho^{(1)}\right]\right]\,\mathrm{d}t'\mathrm{d}t$$

$$= \frac{1}{\hbar^2}\int\limits_{-\varepsilon}^{\varepsilon}\int\limits_{-\varepsilon}^{\varepsilon}\tilde{H}(t)\rho^{(1)}\tilde{H}(t')\,\mathrm{d}t'\mathrm{d}t\,. \tag{S13}$$

When considering again only contributions with the correct phase dependence for the FWM signal ($\sim e^{2i\varphi_2}$) we arrive at

$$\rho^{(2)} = \frac{1}{\hbar^2}e^{i2\varphi_2}\int\limits_{-\infty}^{\infty}\hat{\mathcal{E}}(t)e^{-i\omega t}\sum_{j,v}M_{jv}\left|\mathfrak{T}_v\right\rangle\left\langle\mathfrak{h}_j\right|e^{i\omega_{jv}t}\rho^{(1)}\int\limits_{-\infty}^{\infty}\hat{\mathcal{E}}(t)e^{-i\omega t}\sum_{k,w}M_{kw}\left|\mathfrak{T}_w\right\rangle\left\langle\mathfrak{h}_k\right|e^{i\omega_{kw}t}$$

$$= \frac{1}{\hbar^2}e^{2i\varphi_2}\sum_{j,k,v,w}\hat{s}(\omega-\omega_{jv})\hat{s}(\omega-\omega_{kw})M_{jv}M_{kw}\left|\mathfrak{T}_v\right\rangle\left\langle\mathfrak{h}_j\right|\rho^{(1)}\left|\mathfrak{T}_w\right\rangle\left\langle\mathfrak{h}_k\right|$$

$$= -\frac{i}{\hbar^2}e^{i(2\varphi_2-\varphi_1)}\sum_{i,j,k,u,v,w}\hat{s}^*(\omega-\omega_{iu})\hat{s}(\omega-\omega_{jv})\hat{s}(\omega-\omega_{kw})$$

$$\times M^*_{iu}M_{jv}M_{kw}e^{(i\omega_{iu}-\gamma_{iu})\tau_{12}}\left|\mathfrak{T}_v\right\rangle\left\langle\mathfrak{h}_j\right|\rho^{(0)}\left|\mathfrak{h}_i\right\rangle\left\langle\mathfrak{T}_u\middle|\mathfrak{T}_w\right\rangle\left\langle\mathfrak{h}_k\right|\,. \tag{S14}$$

Finally, taking into account that the emission resulting from a coherence $\left|\mathfrak{T}_v\right\rangle\left\langle\mathfrak{h}_k\right|$ is proportional to $M^*_{kv}e^{(-i\omega_{kv}-\gamma_{kv})t}$, we arrive at the FWM signal

$$\rho^{(\mathrm{FWM})} \sim \sum_{i,j,k,u,v}\hat{s}^*(\omega-\omega_{iu})\hat{s}(\omega-\omega_{jv})\hat{s}(\omega-\omega_{ku})$$

$$\times\rho^{(0)}_{ij}M^*_{iu}M_{jv}M_{ku}M^*_{kv}e^{(-i\omega_{kv}-\gamma_{kv})t}e^{(i\omega_{iu}-\gamma_{iu})\tau_{12}}\,. \tag{S15}$$

Assuming that the spectrum of the laser pulses is much broader than the energy differences between the relevant transitions, we can set $\hat{s}(\omega-\omega_{mn})\approx 1$. We further assume that the initial state is a statistical mixture of the hole states, i.e.,

$$\rho^{(0)} = \sum_n p_n\left|\mathfrak{h}_n\right\rangle\left\langle\mathfrak{h}_n\right|,$$

where we take the thermal distribution for the probabilities p_n. With this the FWM signal can be simplified to

$$\rho^{(\mathrm{FWM})}(t,\tau_{12}) \sim \sum_n p_n\sum_{k,u,v}M^*_{nu}M_{nv}M_{ku}M^*_{kv}e^{(-i\omega_{kv}-\gamma_{kv})t}e^{(i\omega_{nu}-\gamma_{nu})\tau_{12}}\,. \tag{S16}$$

From this we directly find the 2D FWM spectrum

$$\rho^{(\mathrm{FWM})}(\omega,\omega_\tau) \sim \sum_n p_n \sum_{k,u,v} f_{nu}(\omega_\tau - \omega_{nu}) f^*_{kv}(\omega - \omega_{kv}) M^*_{nu} M_{nv} M_{ku} M^*_{kv} \,, \tag{S17}$$

where $f_{iu}(\omega)$ is broadened by γ_{iu} but also takes the spectral broadening by a finite spectral resolution in the detection process into account.

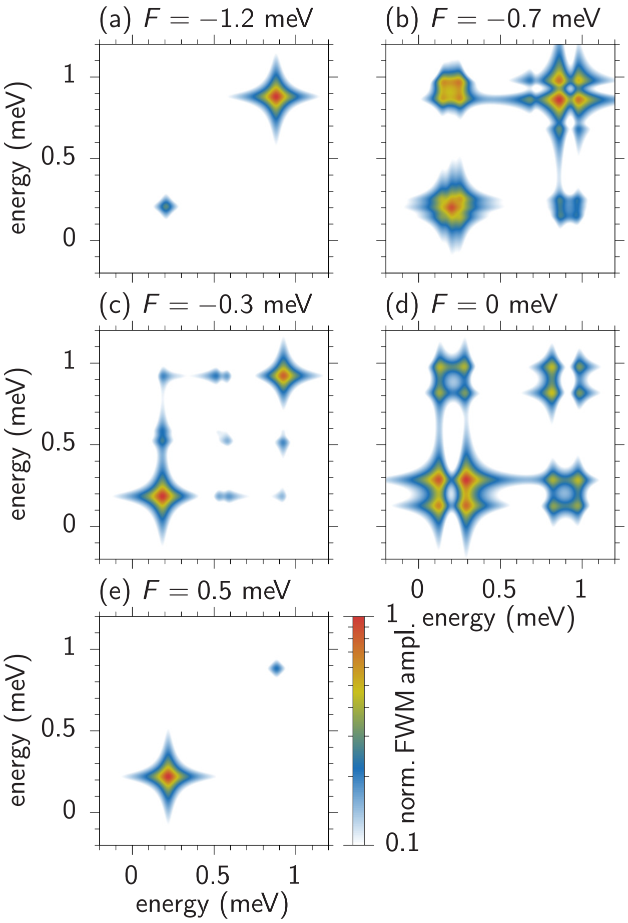

Figure S9: Simulated 2D FWM spectrum using Eq. (S17) for different external electric fields as labeled in each panel.

A few examples for the analytically calculated 2D spectra are shown in Fig. S9 for different bias values as given in the plot. We assume that the spectral broadening is dominated by the instrumental resolution and use the same value as in Fig. 2 in the main text, which roughly agrees with the spectrometer resolution in the experiment. We clearly see that the off-diagonal peaks only appear when the states are efficiently tunnel-coupled in the vicinity of the avoided-crossings in the bias scan (Fig. 2 or S10).

S2C. Dynamical simulations

To simulate the FWM signal while taking a non-vanishing pulse duration into account we calculate the system's dynamics by solving the Lindblad equation for several combinations of pulse phases $e^{i\varphi_1}$ and $e^{i\varphi_2}$.[9,10] We then phase-filter the optically active coherences p in the density matrix by numerically integrating $\int_{\varphi_1,\varphi_2} p e^{-i(2\varphi_2-\varphi_1)} \mathrm{d}\varphi_1 \mathrm{d}\varphi_2$. For a given pulse delay τ_{12} we then determine the FWM spectrum by Fourier transforming the filtered coherence with respect to the real time t after the last arriving laser pulse. The bias scan of the FWM spectrum calculated dynamically is depicted in Fig. S10. We consider a pulse duration (Gaussian standard deviation of the electric field) of 200 fs (this roughly agrees with the full width at half maximum duration of the pulse intensity of 400 fs), a pulse delay of 1 ps, and the previously determined dephasing time of 200 ps. The spectral lines are additionally broadened according to the spectral resolution of the experiment. The most important features, like the pattern of avoided-crossings and intensity distributions agree well with the analytic simulation depicted in Fig. 2 in the main text for a vanishing pulse duration. This shows that the assumption of a flat laser pulse spectrum is a valid approximation to calculate the FWM response.

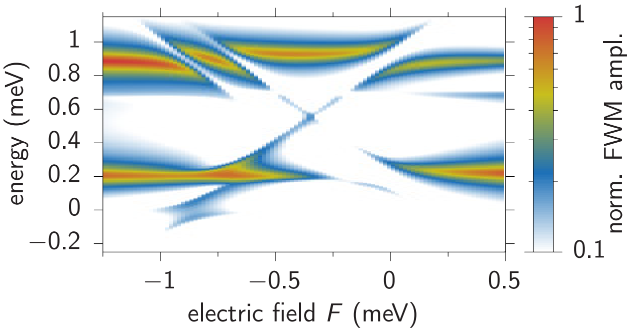

Figure S10: Theoretical bias scan of the FWM spectrum by solving the Lindblad equation and taking into account a non-vanishing pulse duration of 200 fs.

S2D. Inhomogeneous dephasing

In general, inhomogeneous dephasing of a single emitter system originates from fluctuations of the transition energy which happens on timescales slower than a single repetition of the FWM experiment but faster than the repetition rate of the laser. Formally, this situation corresponds to an ensemble measurement. In order to include such slow fluctuations, we assume the transition frequencies ω_{kv}, ω_{nu} in Eq. (S16) to be Gaussian random variables

with mean values $\overline{\omega}_{kv}, \overline{\omega}_{nu}$ and standard deviation σ, which are uncorrelated if $k \neq n$ or $v \neq u$. Upon averaging over the fluctuations, we arrive at

$$\rho^{(\mathrm{FWM})}(t, \tau_{12}) \sim \sum_{n} p_n \sum_{k,u,v} M^*_{nu} M_{nv} M_{ku} M^*_{kv} e^{(-i\overline{\omega}_{kv}-\gamma_{kv})t} e^{(i\overline{\omega}_{nu}-\gamma_{nu})\tau_{12}} f_{\mathrm{deph}}(t, \tau_{12}) \,.$$

where the dephasing factor is

$$f_{\mathrm{deph}}(t, \tau_{12}) = \begin{cases} e^{-\sigma^2(t^2+\tau_{12}^2)}, & k \neq n \text{ or } v \neq u, \\ e^{-\sigma^2(t-\tau_{12})^2}, & k = n \text{ and } v = u. \end{cases}$$

The impact of the inhomogeneous dephasing is shown in Fig. S11, where we show the 2D

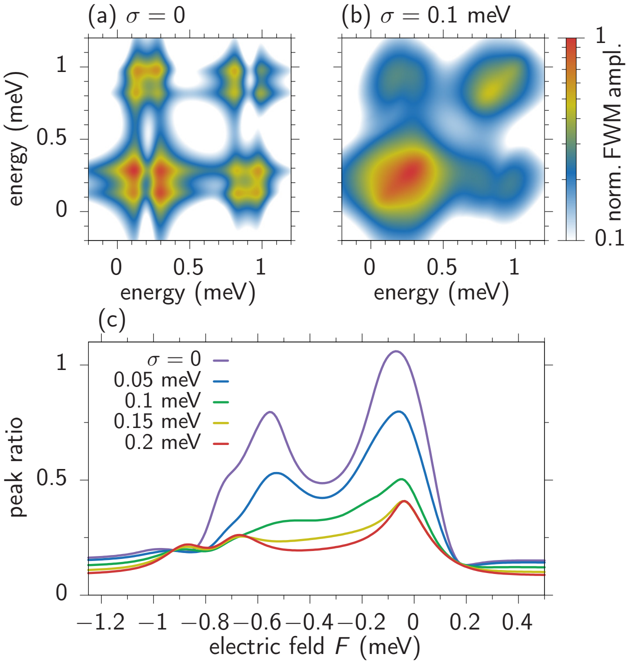

Figure S11: Impact of the inhomogeneous dephasing. (a) 2D FWM with $\sigma = 0$ at an electric field of $F = 0$. (b) Same as (a) but for $\sigma = 0.1$ meV. (c) Simulated peak ratio curves for different inhomogeneous broadenings.

FWM spectrum without inhomogeneous broadening, i.e., $\sigma = 0$ [$F = 0$, same as Fig. S9(d)] in (a) and $\sigma = 0.1$ meV in (b). When presenting the results, we subtract the "background" formed by the power-law tails of the strong diagonal peaks in the spectral area of the off-diagonal ones. We clearly see that the previously sharp peaks get additionally broadened predominantly into the diagonal direction in the 2D spectrum, as well known from several previous works.[11] In Fig. S11(c) we show peak ratios of the form $\sum A_{\text{off-diag}} / \sum A_{\text{diag}}$ as in the main text and increase the inhomogeneous broadening as given in the plot. The values of $A_{\text{off-diag}}$ and A_{diag} are determined by integrating the simulated signal over the areas

shown in Fig. S7(o), with $A_{\text{off-diag}}$ representing the difference of the counting results for the full signal and for the signal with off-diagonal contributions artificially switched off (when only the tails of the strong diagonal peaks contribute). As discussed in the main text, the additional dephasing significantly reduces the visibility of the off-diagonal peaks representing the coherent coupling between the different trion states.

S2E. k·p model

Our $\boldsymbol{k}\cdot\boldsymbol{p}$ model describes a vertically aligned QDM formed by InGaAs in a GaAs matrix. Both QDs are modeled as lens-shaped with the geometry of the bottom QD being set to 18.0 nm in diameter and 4.0 nm in height, while for the top QD it is 22.0 nm and 4.4 nm, respectively. The dots are separated by a distance of 8 nm (base to base) and they are placed on 0.6 nm thick wetting layers (WLs). Note, that all the values are subject to discretization with a mesh adjusted to 0.565325 nm, which is the lattice constant of GaAs at low temperatures. The maximum In content in the dots and in the WL is 45% with a gradual decrease of indium at the edges, which simulates material intermixing. This is done by processing the composition profile with a Gaussian blur with a standard deviation of 0.6 nm.

We calculate single-particle electron and hole states using an eight-band $\boldsymbol{k}\cdot\boldsymbol{p}$ model with the envelope function approximation.[12,13] The strain which arises due to the lattice mismatch of InAs and GaAs is determined by the continuous elasticity approach.[14] The piezoelectric field is included up to the second order in polarization,[15,16] using parameters from Ref.[17] Details of the model are given in Ref.[18] with further improvements of Ref.[19] The trion states are calculated using the configuration-interaction (CI) method, where we consider a basis of 4 electron and 8 hole single-particle states. The electric field is accounted for at the stage of the exact diagonalization of the CI Hamiltonian[20] (with the matrix elements calculated in the basis of single-particle states obtained at zero electric field). The electron-hole exchange is also included at the CI stage. The relevant matrix elements are calculated by mesoscopic expansion of the Coulomb interaction up to second order as in Ref.[21] We then estimate the atomic integrals using rescaled hydrogen-like wave functions[22] with the effective value of the dielectric constant for local (on-site) integrals being equal to 4. The single- and three-particle eigenstates are then used to compute the amplitudes of the optical transitions.[23]

The PL and FWM spectra obtained from the $\boldsymbol{k}\cdot\boldsymbol{p}$ modelling are shown in Fig. S12. Qualitatively, in spite of the much reacher basis, the $\boldsymbol{k}\cdot\boldsymbol{p}$ approach yields the same features in the PL, as the phenomenological model, including the hole and trion resonances as well as the characteristic crossing of transition lines. The FWM spectrum also has exactly the same

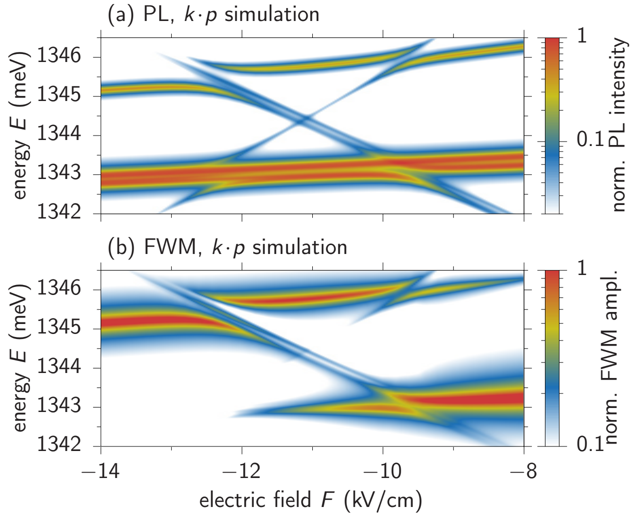

Figure S12: The luminescence (a) and FWM (b) spectra simulated using the system states obtained from the $\boldsymbol{k}\cdot\boldsymbol{p}$ model.

structure, showing essentially two horizontal branches interrupted by avoided-crossings and with intensities decaying in the opposite directions. Quantitatively, the energy scale (which is determined by the Coulomb correlation energies of the three-particle system) is larger in our $\boldsymbol{k}\cdot\boldsymbol{p}$ simulation because of a smaller inter-dot distance, which turned out to be necessary to guarantee the stability of the relevant states in the absence of the AlGaAs barrier in our simplified geometry of the model. One additional feature in the $\boldsymbol{k}\cdot\boldsymbol{p}$ -based PL spectrum is the line at about 1343 meV that crosses all other transition lines without any interaction (avoided-crossing). This line belongs to a transition between states with an excited hole, i.e., electron-hole recombination in the presence of a spectator hole in an excited state (in the p-shell). It is visible in our PL simulation, where all the trion states are assumed to be equally occupied. This is consistent with the measurements shown in Fig. S6, where a similar line becomes pronounced at higher excitation powers. There is no corresponding line in the FWM spectrum, since this is a resonantly excited coherent measurement in which only the lowest hole states are involved.

References

(1) Langbein, W.; Patton, B. Heterodyne Spectral Interferometry for Multidimensional Nonlinear Spectroscopy of Individual Quantum Systems. *Opt. Lett.* **2006**, *31*, 1151.

(2) Kasprzak, J.; Patton, B.; Savona, V.; Langbein, W. Coherent coupling between distant

excitons revealed by two-dimensional nonlinear hyperspectral imaging. *Nat. Photonics* **2011**, *5*, 57–63.

(3) Fras, F.; Mermillod, Q.; G., N.; Hoarau, C.; Schneider, C.; Kamp, M.; Höfing, S.; Langbein, W.; Kasprzak, J. Multi-Wave Coherent Control of a Solid State Single Emitter. *Nat. Photonics* **2016**, *10*, 155.

(4) Hahn, T.; Vaclavkova, D.; Bartos, M.; Nogajewski, K.; Potemski, M.; Watanabe, K.; Taniguchi, T.; Machnikowski, P.; Kuhn, T.; Kasprzak, J.; Wigger, D. Destructive Photon Echo Formation in Six-Wave Mixing Signals of a $MoSe_2$ Monolayer. *Adv. Sci.* **2022**, *9*, 2103813.

(5) Schall, J. et al. Bright Electrically Controllable Quantum-Dot-Molecule Devices Fabricated by In Situ Electron-Beam Lithography. *Adv. Quantum Technol.* **2021**, *4*, 2100002.

(6) Wigger, D.; Karakhanyan, V.; Schneider, C.; Kamp, M.; Höfling, S.; Machnikowski, P.; Kuhn, T.; Kasprzak, J. Acoustic phonon sideband dynamics during polaron formation in a single quantum dot. *Opt. Lett.* **2020**, *45*, 919–922.

(7) Wigger, D.; Mermillod, Q.; Jakubczyk, T.; Fras, F.; Le-Denmat, S.; Reiter, D. E.; Höfling, S.; Kamp, M.; Nogues, G.; Schneider, C.; Kuhn, T.; Kasprzak, J. Exploring coherence of individual excitons in InAs quantum dots embedded in natural photonic defects: influence of the excitation intensity. *Phys. Rev. B* **2017**, *96*, 165311.

(8) Mermillod, Q.; Jakubczyk, T.; Delmonte, V.; Delga, A.; Peinke, E.; Gérard, J.-M.; Claudon, J.; Kasprzak, J. Harvesting, Coupling, and Control of Single-Exciton Coherences in Photonic Waveguide Antennas. *Phys. Rev. Lett.* **2016**, *116*, 163903.

(9) Wigger, D.; Schneider, C.; Gerhardt, S.; Kamp, M.; Höfling, S.; Kuhn, T.; Kasprzak, J. Rabi oscillations of a quantum dot exciton coupled to acoustic phonons: coherence and population readout. *Optica* **2018**, *5*, 1442–1450.

(10) Hahn, T.; Kasprzak, J.; Machnikowski, P.; Kuhn, T.; Wigger, D. Influence of local fields on the dynamics of four-wave mixing signals from 2D semiconductor systems. *New J. Phys.* **2021**, *23*, 023036.

(11) Siemens, M. E.; Moody, G.; Li, H.; Bristow, A. D.; Cundiff, S. T. Resonance lineshapes in two-dimensional Fourier transform spectroscopy. *Opt. Express* **2010**, *18*, 17699–17708.

(12) Bahder, T. B. Eight-band **k·p** model of strained zinc-blende crystals. *Phys. Rev. B* **1990**, *41*, 11992.

(13) Winkler, R. *Spin-Orbit Coupling Effects in Two-Dimensional Electron and Hole Systems*; Springer, 2003.

(14) Pryor, C.; Kim, J.; Wang, L. W.; Williamson, A. J.; Zunger, A. Comparison of two methods for describing the strain profiles in quantum dots. *J. Appl. Phys.* **1998**, *83*, 2548.

(15) Bester, G.; Zunger, A.; Wu, X.; Vanderbilt, D. Effects of linear and nonlinear piezoelectricity on the electronic properties of InAs/GaAs quantum dots. *Phys. Rev. B* **2006**, *74*, 081305(R).

(16) Bester, G.; Wu, X.; Vanderbilt, D.; Zunger, A. Importance of Second-Order Piezoelectric Effects in Zinc-Blende Semiconductors. *Phys. Rev. Lett.* **2006**, *96*, 187602.

(17) Caro, M. A.; Schulz, S.; O'Reilly, E. P. Origin of nonlinear piezoelectricity in III-V semiconductors: Internal strain and bond ionicity from hybrid-functional density functional theory. *Phys. Rev. B* **2015**, *91*, 075203.

(18) Gawarecki, K. Spin-orbit coupling and magnetic-field dependence of carrier states in a self-assembled quantum dot. *Phys. Rev. B* **2018**, *97*, 235408.

(19) Krzykowski, M.; Gawarecki, K.; Machnikowski, P. Hole spin-flip transitions in a self-assembled quantum dot. *Phys. Rev. B* **2020**, *102*, 205301.

(20) Świderski, M.; Zieliński, M. Exact Diagonalization Approach for Atomistic Calculation of Piezoelectric Effects in Semiconductor Quantum Dots. *Acta Phys. Pol. A* **2016**, *129*, 79–82.

(21) Azizi, M.; Machnikowski, P. Interband Coulomb coupling in narrow-gap semiconductor nanocrystals: $\boldsymbol{k} \cdot \boldsymbol{p}$ theory. *Phys. Rev. B* **2015**, *91*, 195314.

(22) Karwat, P.; Gawarecki, K.; Machnikowski, P. Phonon-assisted carrier tunneling with hyperfine-induced spin flip in coupled quantum dot systems. *Phys. Rev. B* **2021**, *104*, 045308.

(23) Gawełczyk, M.; Syperek, M.; Maryński, A.; Mrowiński, P.; Dusanowski, L.; Gawarecki, K.; Misiewicz, J.; Somers, A.; Reithmaier, J. P.; Höfling, S.; Sęk, G. Exciton lifetime and emission polarization dispersion in strongly in-plane asymmetric nanostructures. *Phys. Rev. B* **2017**, *96*, 245425.
